# The Application of High-Resolution, Embedded Fibre Optic (FO) Sensing for Large-Diameter Composite Steel/Plastic Pipeline Performance under Dynamic Transport Loads

**DOI:** 10.3390/s24041298

**Published:** 2024-02-17

**Authors:** Nigel J. Cassidy, Paul O’Regan, Sha Luo, David N. Chapman, Ian Jefferson

**Affiliations:** 1 School of Engineering, University of Birmingham, Edgbaston, Birmingham B15 2TT, UK; s.luo@bham.ac.uk (S.L.); d.n.chapman@bham.ac.uk (D.N.C.); i.jefferson@bham.ac.uk (I.J.); 2 Aquaspira Ltd., Unit 1-1a, Profile Park, Junction St., Colne, Nelson BB9 8AH, UK; po@aquaspira.com

**Keywords:** distributed fibre optic sensing, optical frequency domain reflectometry, pipeline, strain measurement, buried infrastructure, drainage, stormwater

## Abstract

Distributed optical fibre sensing (DOFS)-based strain measurement systems are now routinely deployed across infrastructure health monitoring applications. However, there are still practical performance and measurement issues associated with the fibre’s attachment method, particularly with thermoplastic pipeline materials (e.g., high-density polyethylene, HDPE) and adhesive affixment methods. In this paper, we introduce a new optical fibre installation method that utilises a hot-weld encapsulation approach that fully embeds the fibre onto the pipeline’s plastic surface. We describe the development, application and benefits of the new embedment approach (as compared to adhesive methods) and illustrate its practical performance via a full-scale, real-world, dynamic loading trial undertaken on a 1.8 m diameter, 6.4 m long stormwater pipeline structure constructed from composite spiral-wound, steel-reinforced, HDPE pipe. The optical frequency domain reflectometry (OFDR)-based strain results show how the new method improves strain transference and dynamic measurement performance and how the data can be easily interpreted, in a practical context, without the need for complex strain transfer functions. Through the different performance tests, based on UK rail-road network transport loading conditions, we also show how centimetre- to metre-scale strain variations can be clearly resolved at the frequencies and levels consistent with transport- and construction-based, buried infrastructure loading scenarios.

## 1. Introduction

Over the past decade, distributed optical fibre sensing (DOFS)-based strain measurement systems have become popular for the monitoring and evaluation of infrastructure condition, health, performance and maintenance applications. In the civil engineering sector, modern advances in computational modelling, autonomous data processing techniques and remote monitoring have expanded DOFS use to bridges [1,2], foundations and piles [3,4], pipelines [5,6] and tunnels [7,8]. An extensive review of fibre optic sensing and its application to civil engineering and geotechnical problems can be found in [9] and [10] but it is important to summarise the key practical requirements of any FO-based monitoring technology, particularly with regard to buried infrastructure and, specifically, large-scale drainage and stormwater pipelines. To be effective for long-term use in buried pipeline applications, a DOFS system’s installed optical fibres should:

(i) Be physically robust, durable and suitable for buried use including contact with external backfills, embedment materials and/or internal fluids.
(ii) Be able to withstand a range of natural temperatures (−30 to +50 degrees centigrade), moisture content variations and, potentially, submersion in water/fluids for a long period of time (months or even years).
(iii) Provide accurate, stable, repeatable measurements that can be converted into reliable strain values that are consistent over time and along the fibre’s total installed, active length.
(iv) Be suitable for static and dynamic applications across the typical strain values associated with geotechnical and pipeline applications (i.e., 10 s of microstain through to 10,000–20,000 microstrain) and, therefore, be consistent with conventional strain gauge use.
(v) Be able to measure strain reliably at small (centimetre) and large (>metre) distances along a single FO measurement “cable” of considerable length (10–100 s metres).
(vi) Provide reliable and coherent, time-dependent evaluations of strain under dynamic loading conditions at the typical vibrational frequencies associated with buried infrastructure applications (e.g., road and rail transport, materials handling, construction and pile installation, building works, etc.). These are typically in the range of 0.1–10 s Hz [11].
(vii) Be relatively straightforward to affix (either during or post-installation) using conventional materials and manufacturing/construction techniques by technically competent persons who are not necessarily experts in DOFS applications.

As highlighted in a review of pipeline fibre optic (FO) strain measurement applications [10], the bonding of the fibre optic “cable” to the pipeline is a key element of the measurement system’s performance and although there has been extensive research in the use and performance of different FO measurement techniques, the bonding issue still remains a practical, real-life problem for infrastructure engineers. Due to the sector’s familiarity with strain gauge sensing, adhesives are commonly used to attach the optical fibres to the pipe’s walls (e.g., using cyanoacrylate glues, epoxy resins, etc.). However, research indicates that these bonding methods are not best suited to pipeline applications, particularly plastic pipes. An in-depth study of polyamide-coated fibre optics bonded to aluminium specimens using conventional strain gauge adhesives (i.e., two-part epoxy resins) showed that the transfer of strain from the specimen to the fibre was very much dependent on local strain levels and variations in the elastic properties of the adhesive [12]. The work highlighted that under relatively low levels of strain (<3000 microstrain, με), the performance of the fibre optic strain measurement system was consistent and repeatable with little loss of measurement integrity. However, at slightly higher strains (>3500 microstrain, με) the measurement uncertainty increased significantly with the measured values dropping by approximately 20% after about 30,000 loading cycles. This fatigue-induced deterioration was attributed to weaknesses in the adhesive bond at the base of the fibre and required localised correction factors (or a “strain transfer function”) to be applied to the strain measurement.

Strain transfer functions were initially developed for FO measurements in the late 1990s and early 2000s [13,14,15,16,17] where the strain transfer relationship was deemed to be a function of the properties of the adhesive [18,19] and the geometry of the glued area, i.e., the adhesive thickness, width and bonding length [19,20,21,22]. More recent studies have investigated the detailed nature and geometry of the bonded environment and across a range of glues and epoxy resin types [23,24]. The general conclusion was that the bonding length and thickness variations in the basal adhesive layer have the most significant impact on the transfer function. In addition to the attachment method, the thickness of the fibre optic coating (or cladding layer) also impacts the strain measurement sensitivity with thicker coatings being less sensitive to changes in the substrate strain value [20]. The derived strain function is also dependent on the rheological nature of the coating and the number/thickness of the cladding/coating layers [25,26,27]. Derived strain transfer functions can vary from 1.5–1.1 of the measured strain value and be even less than 1 with particular types of sensing technologies and substrate materials [27]. Slippage between the cladding/coating layers also affects the value and sensitivity of the strain measurement with noticeable measurement “lag” being observed under dynamic loading conditions with multi-layer coatings [28]. Shear deformation under localised strains, within both the coating and adhesive layers, can result in measurement inaccuracies, as can the presence of voids and gaps in the adhesive profile under the fibre [29]. As such, it is important that the fibres are installed in a careful, reliable manner with consistent bonding lengths and uniform adhesive thicknesses.

From a pipeline perspective, the use of thermosetting plastics in drainage pipes introduces a further complication to the strain evaluation process. Plastic drainage pipes are predominately manufactured from polyvinyl chloride (PVC), polypropylene (PP) or polyethylene (PE). However, these materials have low surface energy [30] which means that they are less able to interact with adhesives than the iron, steel, concrete or ceramic materials used in traditional pipelines. As a consequence, voids, bubbles and de-bonded layers can easily form during installation and/or under repetitive loading cycles, particularly with localised stress fields. In principle, surface modification techniques can be used to improve the adhesion properties of the pipe’s materials (e.g., mechanical abrasion, chemical activation paints, etc.) but these complicate the installation process and add further variability to the bonding characteristics of the fibre optic strain measurement system.

**Figure 1:**
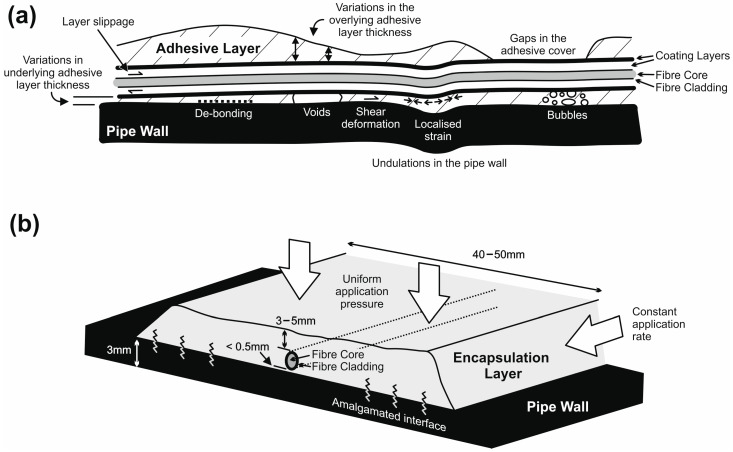
(**a**) Graphical representation of the problems associated with attaching optical fibres to plastic pipelines using conventional adhesive methods (e.g., epoxy resins), (**b**) schematic of the new embedded/encapsulation approach using a hot-weld fabrication technique.

All of these studies show that the operational performance of the fibre-optic-based measurement system can be significantly impacted by the type of optical fibre used, the nature of the bonding approach, the quality of the installation and the material of the pipeline itself. These factors (summarised in Figure 1a) are independent from the fibre optic measurement system but are common for a range of fibre types and application areas.

### Research Aims and Objectives

In this paper, a new method for affixing optical fibres to flexible plastic pipes is proposed where the fibre is completely embedded into the pipe wall structure using a hot-weld encapsulation technique. Our aim is to show how the method can overcome some of the practical limitations associated with adhesive approaches in a relatively simple and cost-effective manner. The approach is novel in that it is the first time that it has been deployed on large-diameter stormwater pipelines and evaluated in a full-scale, dynamic loading test that accurately replicates actual buried infrastructure conditions. The key objective of the research is to provide a real-world performance evaluation of the embedded fibres to de-risk their use and encourage the take-up of the approach across a broad range of buried infrastructure health monitoring applications. In doing so, we hope that the research will provide practical reference and reliable information for engineers to compare and contrast the results to their own FO-based strain measurement applications.

In the Materials and Methods section, the development of the embedment approach will be described in more detail including a summary of the preliminary performance tests carried out in collaboration with the pipeline manufacturer. Following on from this, the practical behaviour of the fibres is evaluated in detail as part of a comprehensive “Below Ground” trial conducted on a buried stromwater drainage structure fabricated from 1.8 m diameter, composite, steel–high-density polyethylene (HDPE), spiral-wound pipes manufactured by Aquaspira Ltd., Nelson, UK [31]. The trial involved the sequential loading of the pipeline structure in a simulated rail/road transport scenario where the testing programme consisted of over 1,000,000 cycles of 1–11-Tonne dynamic loads (8 Hz and 1 Hz) plus 40-Tonne static loading. The trial replicated the equivalent of more than 7000 “train pass loads” on the UK main rail network and included measuring soil pressure and displacement, pipeline displacement, discrete linear stain (strain gauges) dynamic and static FO-based distributed strain (using an optical frequency domain reflectometry (OFDR) system) and actuator loading/extension before (pre), during (syn) and after (post) each loading cycle.

In the results that follow, emphasis will be placed on the performance of the dynamic FO-based strain measurements as these are the most relevant to the aims of the paper. However, the findings are equally relevant to the static FO measurements conducted during the trial. A more detailed account of the pipeline performance aspects of the trial can be found in [32] while the full trial results, including all recorded data, are the subject of a forthcoming case study report publication currently in preparation [33]. Finally, the paper will conclude with a discussion on the benefits and drawbacks of using the embedded fibre approach and illustrate the quality of the results that can be obtained during dynamic loading applications.

## 2. Materials and Methods

### 2.1. Preliminary Performance Tests of Different Attachment Methods

To meet the challenging demands of plastic stormwater pipeline applications, a number of different attachment approaches were investigated with physical tests conducted on primitive HDPE samples, test specimens of singular pipe profiles and fully constructed pipe. These included specialist epoxy resins (cold and hot applications), activated glues, adhesive tapes, thermosetting glues and the proposed hot-weld encapsulation method. In each case, the affixed optical fibres were subjected to repeat bending stresses, external pressure/impact and submersion in water. All of the adhesive approaches suffered from the issues highlighted in the Introduction and, more importantly, were prone to de-bonding even after moderate bending stresses were applied. As such, the hot-weld encapsulation method was selected as it had the advantages of:

- Fully covering the optical fibre with a 3–5 mm thick, 40–50 mm wide layer of HDPE (Figure 1b). This protected the fibre from physical damage and contact with water and isolated it from local extremes of temperature change. It also facilitated the use of polyimide-cladded, singular (monofilament) optical fibres that do not suffer from the same intra-layer slippage effects observed in multi-coated fibres.
- Amalgamating with the HDPE pipe wall to produce a fully bonded, mechanically continuous basal interface; therefore, completely encapsulating the fibre in an embedded installation. This improved the continuity of strain transfer from the pipe wall to the fibre and negated the de-bonding issues prevalent with adhesive approaches.
- Being applied using conventional manufacturing tools and equipment (e.g., weld guns) either during the pipeline’s manufacture or pre-/post-installation on site (i.e., a practical and cost-effective solution). The use of conventional weld guns allowed the fibres to be installed in between the ribs of the composite spiral-wound pipe (external fibres) or internally on the smooth but undulating surface of the pipe wall (the “water-wall”).

**Figure 2:**
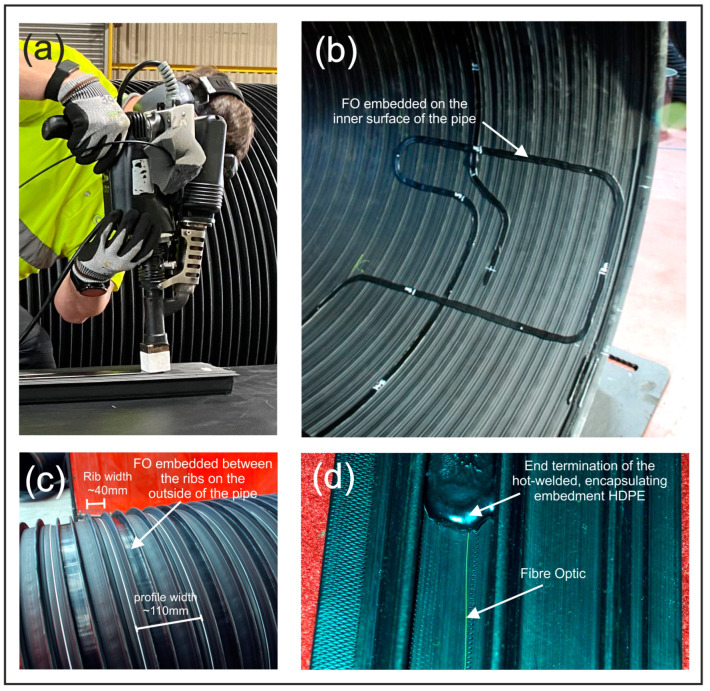
(**a**) The HDPE hot-weld encapsulation and embedment approach being applied to a test specimen of HDPE composite spiral-wound stormwater pipe profile, (**b**) Completed installation of the embedded optical fibre on the internal “water-wall” surface of the pipe—note the undulations in the otherwise smooth surface, (**c**) Completed installation of the embedded optical fibre on the outside of the stormwater pipe, (**d**) Close-up of the fibre and hot-weld layer.

After some trial and error, an optimised fabrication method was developed that applied the HDPE to the pipe wall at 150–180 °C using uniform application pressures (hand pressure) and a constant application rate of ~1 cm/s for the weld. A 35 mm wide, continuous-feed (at 0.5 g/s), temperature-controlled, hot-weld gun was used to apply the encapsulating HDPE layer with the optical fibre held under slight tension on the surface of the pipe. Figure 2 shows the hot-weld method being used on a test specimen (Figure 2a), the embedded fibre between the composite pipe’s ribs (Figure 2c), the fibre installed on the internal, water-wall surface of a pipe (Figure 2b) and a close-up of the fibre and hot-weld layer (Figure 2d).

The temperature of the hot-weld HDPE layer (applied at 150–180 °C) was selected to keep it below the manufacturer’s maximum 220 °C operating temperature for the fibre (i.e., to avoid physical damage) but also to ensure that the HDPE had a low enough viscosity to guarantee flow in and around the fibre at the point of application. As the HDPE cools (approximately 20–30 min to reach room temperature) it contracts slightly, “clamping” the HDPE around the fibre and guaranteeing close coupling between the fibre and the encapsulating layer. The uniform application pressure pushes the fibre up against the pipe wall with post-installation inspection showing that the technique produced a surprisingly constant, below-fibre, basal layer of less than 0.5 mm along the whole length of the installation. Note the regular width of the encapsulation layer on the inside of the pipe on the undulating, but smooth, inner water-wall surface (Figure 2b). Also note the rib and profile width of the manufactured spiral-wound pipe (~40 mm and ~110 mm, respectively—Figure 2c). These features will have an influence of the local variation of stress and strain along the pipe when loaded as they will subtly alter the centimetre-scaled value of stiffness in the wall of the pipe.

### 2.2. Baseline Strain Evaluation of the Encapsulation and Embeddment Process

One disadvantage of the hot-weld encapsulation method is that the HDPE must be applied under pressure, albeit at relatively low levels (i.e., hand pressure). In addition, the fibre is held under slight tension on the surface of the pipe. This introduces a non-zero, post-manufacturing strain into the embedded fibre that needs to be calibrated out prior to pipeline loading. The undulating, inner surface of the pipe introduces further local permanent strains that, in extreme cases, could significantly reduce the measurement range of the FO system. To test this, two identical, pre-calibrated strain sensor fibres (Luna HD6S series) were installed on the inside of an unloaded pipe using the hot-weld encapsulation method and cyanoacrylate glue. Temperature-compensated strain values were obtained from each sensor using a Luna ODiSI 6100 series optical frequency domain reflectometry (OFDR) measurement system [34]. The system utilises swept-wavelength interferometry (SWI), Rayleigh-based backscatter amplitude measurements to determine the strain along the optical fibre at incremental distances (gauge pitch) of 0.65, 1.3, 2.5 or 5.2 mm and measurement frequencies of up to 25–100 Hz, depending on sensor length/pitch. A pitch length of 1.3 mm was selected for the tests as it provided a maximum measurement frequency of 25 Hz but was small enough to pick up the subtle, sub-centimetre variations in strain produced by undulations in the water-wall of the pipe. The manufacturer’s quoted accuracy for the system in this configuration was: measurement uncertainty, ±4 microstrain (με); system accuracy (sensor and measurement system), ±30 microstrain (με). Further information on the Luna ODiSI 6100 (Luna Innovations, Camberley, UK) measurement system used in this research can be found in [34] with the relevant specifications provided in Table 1.

**Figure 3:**
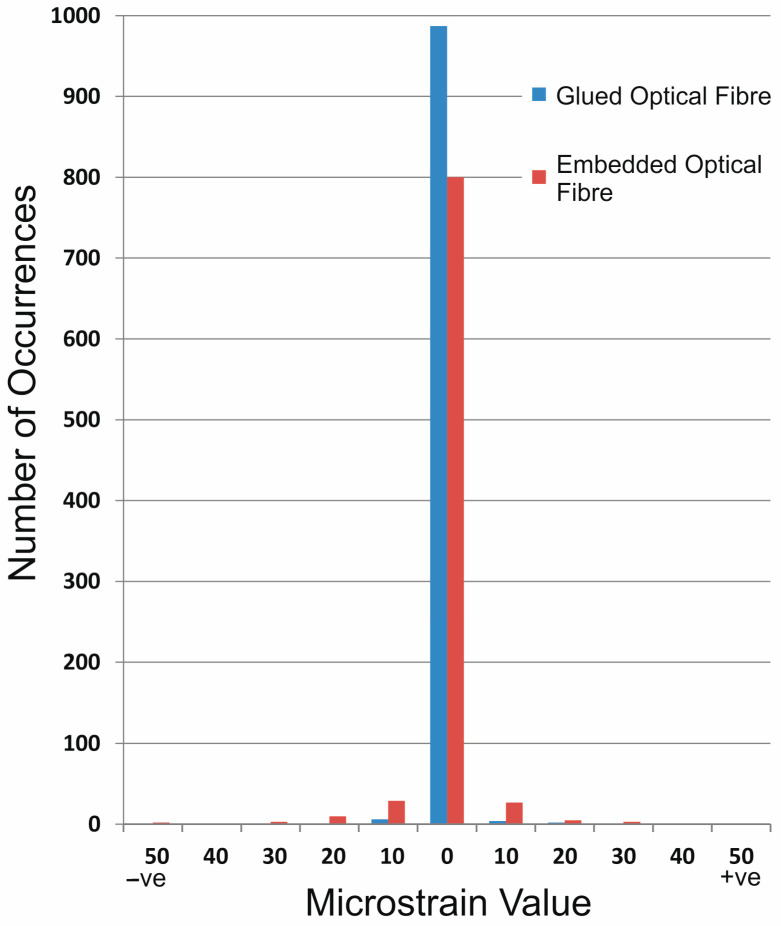
Distribution of unloaded strain measurements for the glued (blue) and embedded (red) fibre optic sensors installed on the inside of a composite stormwater drainage pipe. The data show the mean of 30 repeat measurements at each of 1000, 1.3 mm gauge pitch increments.

Data were collected along a 1.3 m length of each sensor (1000 gauge pitch increments) and averaged across 30 repeat measurements. Complementary measurements were made on an identical length of un-affixed sensor with a resultant, overall system accuracy of ±6 microstrain (με), which is the base level of system accuracy to be expected in all tests and an improvement on the manufacturer’s quoted figures. The distribution of unloaded strain measurements is shown in Figure 3 for the glued (blue) and embedded fibres (red). For the glued fibre sensor, it is clear to see that the majority of the measurements (>96%) are within the ±0–5 με interval and the rest less than ±15 με. For the embedded fibre, the results are similar with the majority (80%) of recorded measurements in the ±0–5 με interval and the rest having a slightly wider distribution but still below the ±35 με level. The narrow distribution of the embedded data illustrates that despite having pressure applied to the fibre as it is encapsulated, the resultant strains are low enough (i.e., pretty much within in manufacturer’s stated system accuracy) to make a negligible difference to the measured results. Note, however, that 121 of the 1000 mean measurements were recorded as “not a number” (NaN) by the measurement system. These data gaps are a consequence of local strain gradients that cannot be resolved by the calibrated sensor. They only represent 1.2% of the mean measurements but their presence means that post-collection data processing is required to complete the data set (as discussed in more detail in the following sections).

### 2.3. Strain Measurement Evaulation under Controlled Condition—The “Above Ground” Trials

To further assess the operational performance of the embedded optical fibres, an extensive programme of commercial trials was undertaken in collaboration with the pipeline manufacturer, Aquaspira Ltd. The trials included “Above Ground” loading tests on an unrestrained, 5.2 m long, 1.8 m diameter composite stormwater pipe (2 × 2.6 m long coupled sections). The loading test included repeat sequential point and linear crown loadings of up to 10 Tonnes. Dynamic and static FO-based strain measurements were collected linearly along the inside of the crown (i.e., the top of the pipe) and circumferentially around the pipe at ~1.5 m intervals along its length. Dynamic DOFS data were collected with the Luna ODiSI 6100 system and pre-calibrated sensors as described previously, whilst the static DOFS data were collected with a Luna optical backscatter reflectometer (OBR) 4600 series system [35] using the same type of optical fibre as the dynamic measurement system. The FO-based data were complemented by strain gauge and pipe displacement measurements [36]. For brevity and conciseness, the data are not replicated here but the trial showed that the embedded, fibre optic strain measurement system produced results that were:

- Consistent, repeatable and reliable for strain values up to 10,000 microstrain for both the dynamic and static measurement systems.
- Typically, within ±5% of the strain gauge measurements where the measurements were co-located on the pipe.
- Consistent with regard to temperature variations and at accuracies that matched the manufacturer’s specifications.

The findings from the tests provided the confidence that the new embedded fibre approach will produce results that, from a practical perspective, will not require the use of complex transfer functions to provide reliable and accurate strain evaluations. This was considered a significant advantage over adhesive installation approaches and led to the development of the “Below Ground” full-scale, dynamic loading test programme described in the following section.

## 3. Results from the “Below Ground” Full-Scale, Dynamic Loading Trial

**Figure 4:**
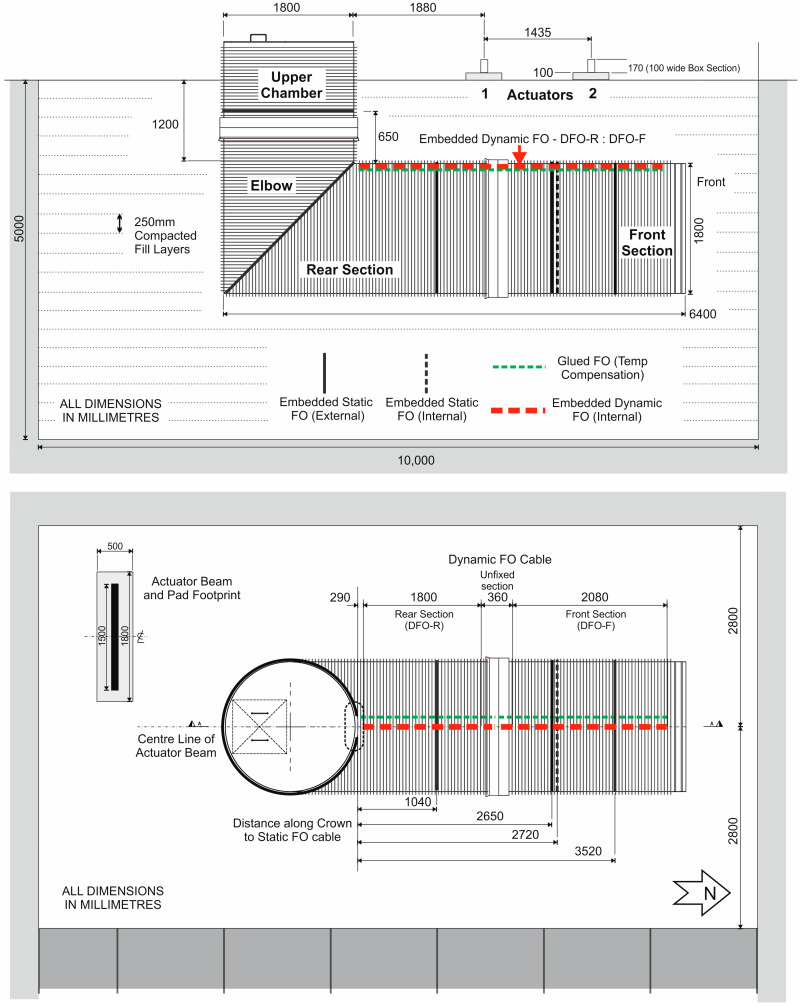
Cross-section and plan of the “Below Ground” loading performance trial conducted on the buried Aquaspira stormwater pipeline and fabricated chamber structure.

To illustrate the performance and applicability of the embedded optical fibres in practical circumstances, the pertinent results from a comprehensive “Below Ground” full-scale, dynamic loading trial are presented that accurately replicate the effect of long-term cyclic transport (road/rail) loads on a buried stormwater pipeline structure. The trial, conducted at the National Buried Infrastructure Facility (NBIF) at the University of Birmingham in late 2022, consisted of repeat cyclic loading tests (1–11 Tonnes at 8 Hz and 1 Hz) over a buried, 1.8 m diameter stormwater pipeline structure with a connected access chamber. Buried to a cover depth of 1.2 m (i.e., the standard “minimum” depth of cover for this diameter/type of pipe), the pipeline was subjected to over 1,000,000 individual cyclic loads from two hydraulic actuators spaced at a distance consistent with the UK’s standard rail network track gauge (i.e., 1.435 m). Figure 4 illustrates the layout and plan of the buried pipeline structure, the location of the installed dynamic and static optical fibres and the position of the two hydraulic actuators.

**Figure 5:**
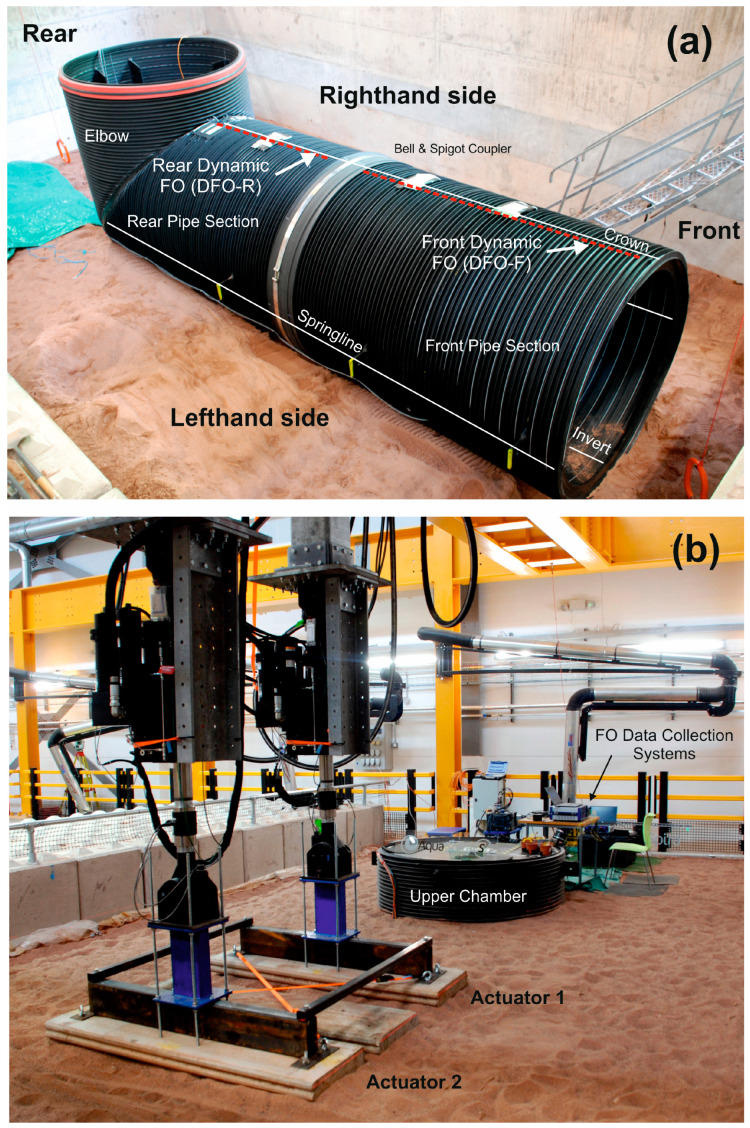
(**a**) Image of the pipeline and elbow components of the stormwater pipeline structure before burial (taken from the lefthand side)—note the location of the dynamic FO and bell and spigot connector, (**b**) Image of the “Below Ground” trial set up (taken from the righthand side) including the hydraulic actuators, buried stormwater pipeline structure (upper chamber in view) and data collection instrumentation.

The NBIF “pit” was 5 m deep, 10 m long and 5.6 m wide and filled with 250 mm thick compacted layers of moderately sorted, coarse-sand-grade backfill of quarried sand around the pipe to a density of ~1650 kg/m^3^ and with a moisture content of 7–10%. The trial included temperature-compensated dynamic and static DOFS measurements using the Luna ODiSI and OBR systems described previously as well as soil pressure/displacement, linear strain and pipeline displacement measurements collected during each loading test. The buried stormwater pipeline consisted of three components. A 2.6 m long Front Section with a blocking end plate at its front face, which was connected to a 3.8 m Rear Section and 90° elbow fabricated from 1.8 m diameter, composite, steel-reinforced, spiral-wound, HDPE Aquaspira pipe. An Upper Chamber section, fabricated from the same type of pipe, completed the structure and extended above the surface by approximately 600 mm. The three components were connected by Aquaspira’s standard bell and spigot connector (Figure 5).

The optical fibres were installed in the pipeline structure before burial (Figure 5a) and measurements were taken after burial once the loading system was fully tested for operational safety. The pre-calibrated dynamic optical fibre sensor was installed inside the pipeline in two segments, one in the front section (2080 mm long) and the other in the rear (1800 mm long). A 360 mm section of the sensor was left “unfixed” across the bell and spigot connector to avoid any potential local extremes in strain building up in the fibre due to movement across the connector during burial and compaction. In hindsight, this precaution was unnecessary as the pipe did not move to any significant degree and the sensor could have been embedded across the connector with no loss of measurement integrity.

The results from four separate loading test scenarios are shown to illustrate the quality of the measured data and how the new embedment method can be used to easily obtain detailed strain/deformation information on the pipeline structure. For conciseness, only the data from the dynamic DOFS measurement system are shown as the same installation approach was used on all optical fibres. In addition, the data from both systems showed very similar characteristics in terms of measurement accuracy, reliability and overall performance. However, the static system’s data did not suffer from data gap issues, the consequences of which are considered later in the paper. The selected tests are:

(1) Post-installation and post-burial but before the loading cycles were conducted. To illustrate the absolute, pre-loaded baseline level of strain across the pipeline as a result of backfill compaction.
(2) Synchronous loading of both actuators at 8 Hz with 1–11-Tonne sinusoidal, cyclic loads. To illustrate the syn-loading change in strain across the pipeline. Additional data from a 1 Hz asynchronous cyclic load test are also provided to illustrate the spectral response of the installed fibres and measurement system.
(3) Cyclic loading of one actuator at 8 Hz with 1–11-Tonne sinusoidal, cyclic loads. To illustrate the change in syn-loading strain pattern across the pipeline with only one actuator in operation.
(4) Static loading of both actuators with a 40-Tonne total load. To illustrate the maximum syn-loading strain pattern across the pipeline under a static, constant load.

In each instance, the illustrated strain data are the mean value of 30 s of measurements collected at 25 Hz along the full active length of the fibre. As with previous tests, 1.3 mm pitch gauge increments were chosen in order to pick up any sub-centimetre strain changes due to movement of the pipe’s ribs. The data have been de-spiked (anomalously high values interpolated between adjacent measurements—typically less than 10 points across 3500 measurement increments) and “NaNs” filled in with linear or spline interpolation. The interpreted displacement of the pipeline at its crown is also shown to illustrate the direction of deflection movement. Although not shown for clarity, these displacements are consistent with the independently measured, crown-to-invert deflections recorded in the front section of the pipeline.

### 3.1. Post-Installation, Pre-Loading Results

**Figure 6:**
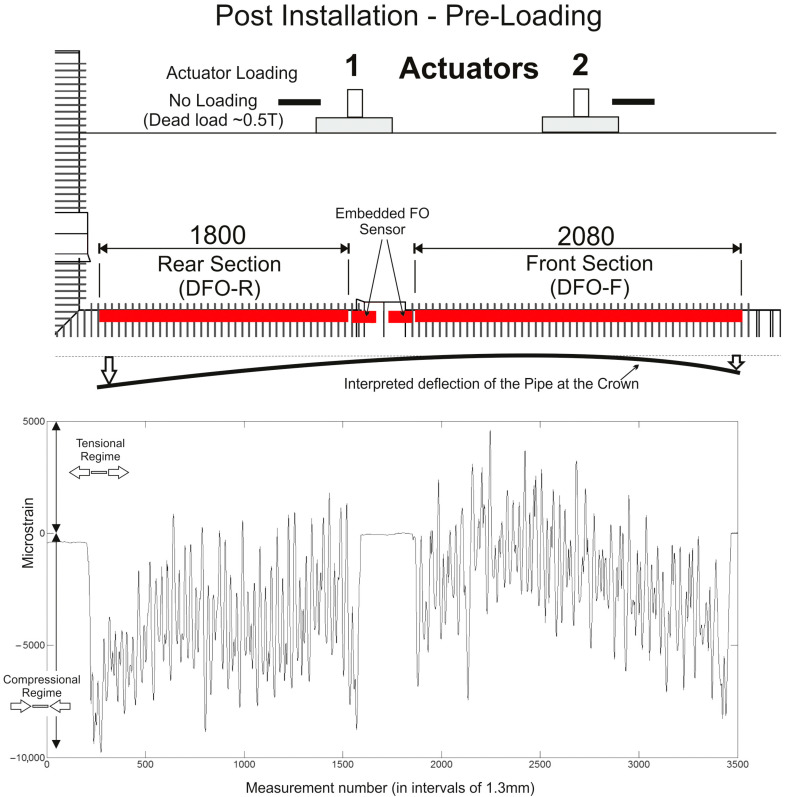
Dynamic strain data results for the post-installation, pre-loading condition across the front and rear sections of the embedded fibre optic sensor (data shown as the mean of absolute measured values taken across a 30 s “snapshot” data collection period). Positive values are tensional strain and negative values compressional strain along the fibre. The black line illustrates the interpreted pipeline deflection.

Figure 6 shows the results from data collected post-installation but ahead of all loading tests. The dynamic system’s strain data are shown as a “snapshot” with the mean of the measured absolute values in 1.3 mm pitch gauge increments. The effect of backfill compaction can be seen in the data with the strains increasing from the “stiffer” center of the pipeline, where the bell and spigot coupler is situated, out to the more flexible ends of each pipeline section.

It is important to note that the absolute strain value ranges from an average of about −100 με through to about −7000 με, indicating that the fibre is under compression along its full length (−ve values are compressional strain in this case). With a maximum specified strain of ±15,000 με for the calibrated sensor, this illustrates how significant permanent strains can potentially limit the range of any FO-based measurements. Peak values are close to −10,000 με which could also limit the maximum relative change in measured strain under load to about 5000 με.

### 3.2. Synchronous Loading, Syn-Loading Results—Change in Strain Value

**Figure 7:**
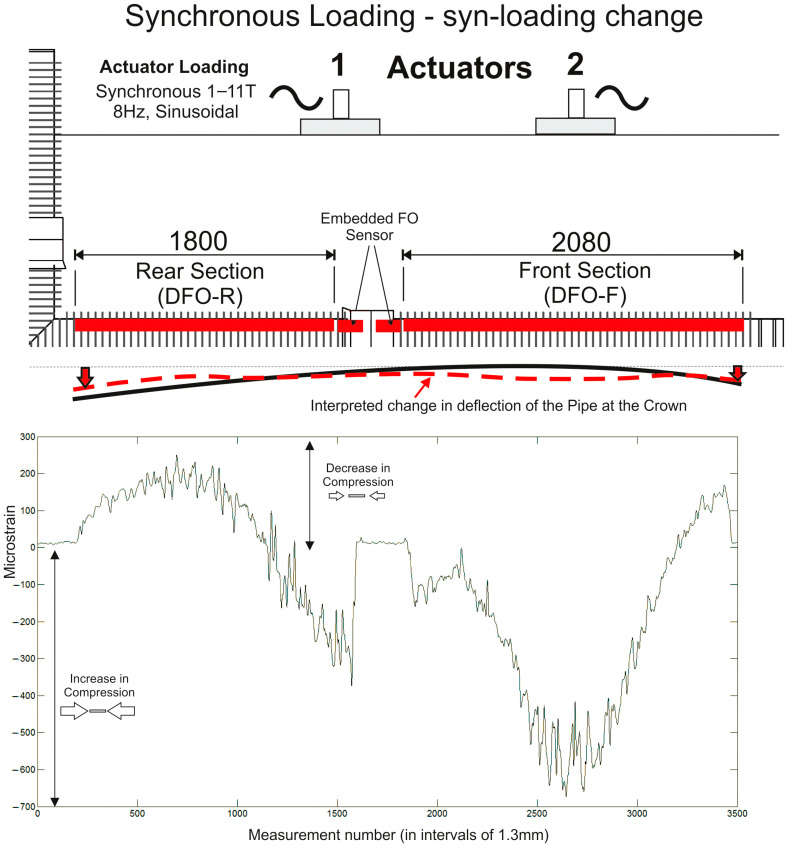
Dynamic strain data results from the front and rear sections of the embedded fibre optic sensor for the synchronous loading test (data shown as the mean of measurements taken across a 30 s “snapshot” data collection period). The data were collected during the loading cycle (syn-loading) and are shown as the change in strain (+ve values are a decrease in compressional strain and −ve values an increase in compressional strain along the fibre). The dashed red line illustrates the interpreted change in pipeline deflection.

Figure 7 shows the results from data collected during the synchronous loading tests during the active loading cycle. The actuators were synchronously cycled at 8 Hz, in tandem, with a sinusoidal, load-limited cycle. The actuators were not extension-limited during the tests, which allowed the buried materials to compact in a natural and unconstrained manner. This approach was taken to provide the most realistic replication of real-world transport loading scenarios. The strain data are shown as the change in strain between the pre- and syn-loading conditions where positive values are a decrease in compressional strain and negative values an increase in compressional strain along the fibre.

The displayed strain values are the mean of 1.3 mm pitch gauge increments and are “smoothed” as a moving average across seven increments (i.e., 9.1 mm). This was carried out to decompress the data across increments at a length that is consistent with the installed linear strain gauges (10 mm) and visually improve the form of the data for interpretational purposes. The effect of the increased loads can be seen in the elevated value of compressional strain in the fibre around the footprint of the actuators, with the bell and spigot coupler providing a noticeably stiffer response to the load directly above it. Interestingly, with the loads situated towards the front end of the pipeline, the rear end of the fibre is now under reduced compressional strain indicating a slight “upwards” deflection in the pipeline’s crown at this point. The additional, relative change in strain is less than 700 με, well within the maximum range limit of the measurement system.

**Figure 8:**
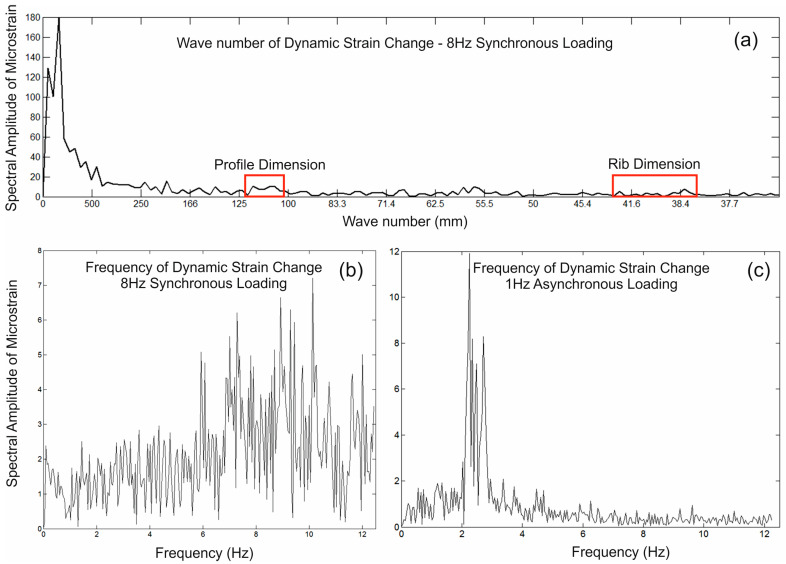
Spectral analysis of the dynamic strain data collected during the loading test. (**a**) Wavelength/wavenumber of the data collected along the full length of the fibre—synchronous loading at 8 Hz, (**b**) Frequency component of the data collected at measurement point 550—synchronous loading at 8 Hz, (**c**) Frequency component of the data collected at measurement point 550—asynchronous loading at 1 Hz.

Figure 8 shows the spectral characteristics of the data with Figure 8a illustrating the spectral wavelength or wavenumber of the collected data, which represents the spatial dimensions of strain change across the pipe.

As would be expected, the dominant change is associated with distances corresponding to the pipeline’s individual section lengths (>1 m). However, there are, arguably, weak but noticeable spectral signatures at ~110 mm, ~60 mm and from 38–42 mm. Two of these coincide with the rib (40 mm) and profile (110 mm) dimensions and the 60 mm peak is consistent with the mid-gap distance across two ribs. Although not exactly dominant, these shorter wavelength signatures indicate that the embedded fibre is picking up subtle, local dynamic changes in strain associated with the constructed form of the pipeline, an important aspect for condition monitoring. Figure 8b shows the frequency component of the time-dependent data collected at measurement point 550 during the synchronous loading at 8 Hz (data collected at a 25 Hz sampling frequency and for a total of 30 s). Although the dominant frequency of the loading is lower than the 25 Hz measurement frequency, the spectral response is spread around the 7–11 Hz range with no real dominant peak. The sensor manufacturer’s information defines the maximum dynamic loading frequency for this set up as 2–5 Hz (not 25 Hz) which suggests that the spectral response is, in practice, dependent on the sensor’s calibration “resolution” across a number of increments. Typically, a minimum of five points per full sinusoidal cycle are required to accurately reconstruct a cyclic signature in digitised data. This would fit with the observed data where the contribution from strain changes above 5 Hz is less accurately represented. Although the spectrum is a little incoherent, there is a distinct change in spectral amplitudes from above 7 Hz (i.e., a general increase), which allows for some meaningful comparative interpretations to be made, even at higher frequencies. The spectral sensitivity at lower frequencies is illustrated in an additional analysis from equivalent data collected during an asynchronous loading test (Figure 8c) where both actuators were operated at 1 Hz but moving in opposing directions (i.e., flip-flopping). As such, the loads at the surface were cycling at a rate of 2 Hz, which can be clearly seen in the spectral response.

### 3.3. Cyclic Loading of One Actuator and Static Loading of Both Actuators—Change in Strain Value

**Figure 9:**
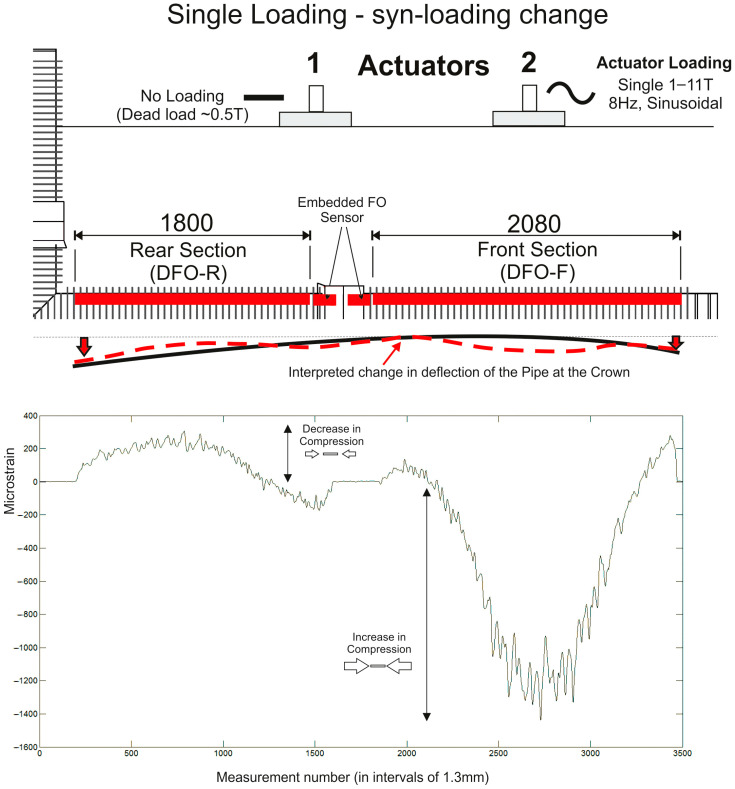
Dynamic strain data results from the front and rear sections of the embedded fibre optic sensor for the single actuator loading test at Actuator AC2 (data shown as the mean of measurements taken across a 30 s “snapshot” data collection period). The data were collected during the loading cycle (syn-loading) and are shown as the change in strain (+ve values are a decrease in compressional strain and −ve values an increase in compressional strain along the fibre). The dashed red line illustrates the interpreted change in pipeline deflection.

**Figure 10:**
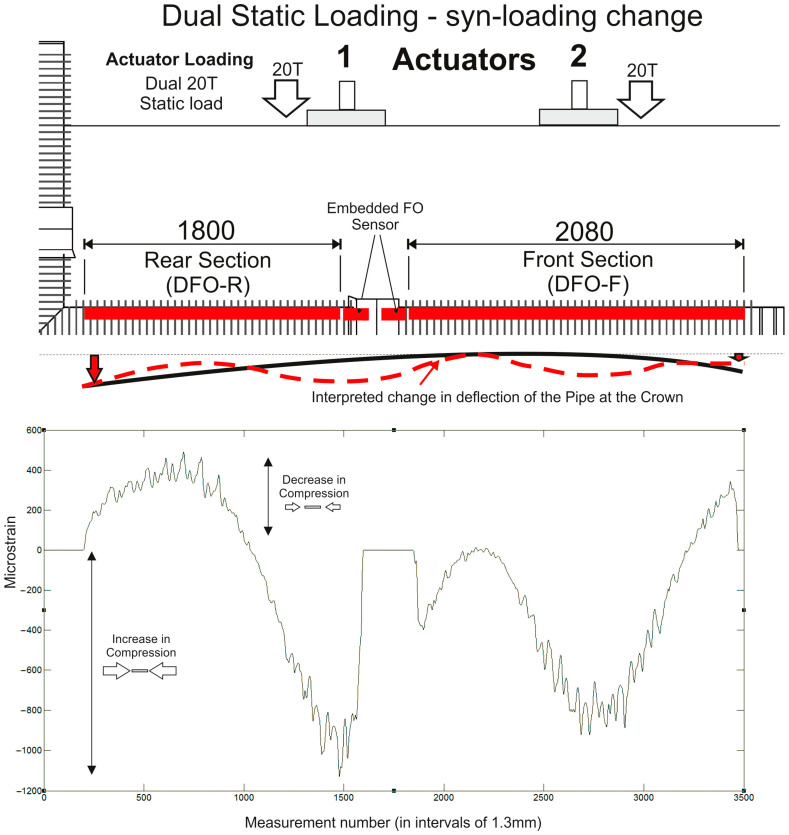
Dynamic strain data results from the front and rear sections of the embedded fibre optic sensor for the dual actuator static loading test with a load of 2 × 20 Tonnes (data shown as the mean of measurements taken across a 30 s “snapshot” data collection period). The data were collected during the loading cycle (constant static loading from both actuators) and are shown as the change in strain (+ve values are a decrease in compressional strain and −ve values an increase in compressional strain along the fibre). The dashed red line illustrates the interpreted change in pipeline deflection.

In the final two examples, the change in strain profile along the pipeline is illustrated as a consequence of different loading regimes. Figure 9 shows the change in strain associated with 1–11-Tonne 8 Hz loading on one actuator only (AC2 at the front section of the pipeline structure). Figure 10 details the broader loading response of the whole pipeline under 2 × 20-Tonne (40 Tonnes in total) constant, static loads.

In both cases, the strain profile follows a predictable pattern with peak strains centered under the actuators at maximum amplitudes of (−ve) 1000–1200 με, well within the maximum allowable strain. Interestingly, the local-scale strain value similarly increases and decreases with loading change (within 100–200 με) but retains the same distribution pattern along the length of the fibre. This illustrates how well the embedment approach is able to transfer local variations in strain from the pipe to the fibre without measurement lag or loss of data integrity.

## 4. Discussion

The loading tests conducted as part of the Below Ground trial, along with the preliminary data from the Above Ground trial, clearly show that the embedment approach can produce coherent, reliable and repeatable strain measurements across a range of loading scenarios. The pattern, amplitude and spectral response of the data are consistent at a range of temporal and spatial scales, allowing for detailed interpretations to be made on the pipeline’s deformation at rib/profile (centimetre) and sectional (metre) distances. The loading distribution and spatio-temporal changes are clearly resolved and the real-world nature of the trial means that the embedment approach can be used in confidence under loading scenarios that are routinely observed with buried drainage infrastructure. The practical issues with adhesively bonded fibres have been negated and the ability to incorporate monofilament optical fibres means that the cost and complexity of the installations are reduced. Being able to install the fibres with conventional fabrication tools and methods also provides a practical benefit in that the embedment process can occur either before or after pipeline installation.

One potential disadvantage of the embedment approach is the presence of NaNs, or gaps, in the recorded strain data (which can also occur in adhesively bonded data). These require post-collection data analysis, editing and interpolation to produce a full data set. The gaps appear to be associated with high strain gradients at localised points where it is believed that the pre-calibrated optical fibre sensor is unable to reconstruct a coherent value of strain from the recorded backscatter signals. The thoughtful selection of sensor gauge pitch and temporal sampling rate can reduce the number of NaNs, as can an increase in the number of time increments included in each averaging cycle. However, these gaps can never be completely eliminated and, although they typically constitute less than 15% of the processed data, some form of data interpolation will always be necessary between adjacent coherent values. It is pertinent to note that the OBR-based measurement system (i.e., the static strain measurement system) did not produce data gaps in the recorded strain values. The OBR approach does not use pre-calibrated fibre sensors but instead relies on independent instrument calibration and a pre-determined formula to convert the backscatter signal to a strain value. However, it produces data that are always relative to some nominal baseline measurement rather than giving independent, absolute strain values. This suggests that the NaN issue is ultimately related to instrument/calibration complexities with the dynamic measurement system rather than any particular issue with the embedment/attachment method. An analysis of the practical impact of gaps in the data and the cause of high-strain-related NaN recordings is the subject of further experimental work.

The total encapsulation of the monofilament, singularly cladded optical fibre means that it is protected from external damage and provides “tight” strain transfer coupling between the pipe and the fibre. It overcomes the issues observed with cladded/coated fibres (i.e., intra-layer slippage) and results in a much simpler relationship between the real and measured strain. The close correlation between the independent strain gauge values and the fibre-based measurements observed in the Above Ground tests highlights the reliability and accuracy of the strain measurements and, from a practical perspective, means that reliable evaluations of strain change can be obtained without the need for complex strain transfer functions. Nevertheless, the monofilament fibres are quite fragile and easily “snapped” if mishandled. That said, they are quite robust under tension and can be curved into bend radiuses of ~100 mm without subsequent data issues. When carefully installed by proficient, skilled operatives, their fragility is not an issue but it does take time and planning to ensure that the embedded fibre runs are installed competently and remain intact. When encapsulated, the fibres are very robust and recent physical tests (point impacts and extreme bending/twisting of the pipe walls) have shown the embedment process appears to spread the stresses across and along the weld bead, resulting in less localised extremes in strain on the fibre. Even with strains on the pipe wall exceeding 30,000 με (estimated value), the embedded fibres did not appear to suffer any physical damage and remained coherent and intact along their length.

## 5. Conclusions

The findings of this research show that the newly proposed embedment approach is perfectly capable of providing reliable distributed optical fibre strain measurements that can be used for the condition and health monitoring of buried plastic/composite stormwater pipeline infrastructure. The robustness and dependability of the embedded optical fibre installation approach have been demonstrated with full-scale, buried trials where a composite HDPE–steel stormwater pipeline structure and embedded fibres have been subjected to a range of loading scenarios consistent with major UK transport (road/rail) infrastructure environments. However, the proposed embedment approach does require careful installation by diligent, skilled operatives to guarantee fibre integrity. In addition, data gaps can become an issue when using pre-calibrated FO sensors and, as a consequence, some post-collection data processing will always be required to ensure that the data sets are complete. This is the first time that such an installation approach has been assessed under real-world conditions and provides the necessary practical confidence for asset owners, civil engineers and geotechnical professionals to adopt the method for their own infrastructure health monitoring applications. Further work will focus on (a) widening the applicability and relevance of the approach to other thermosetting plastics (e.g., PVC) and pressurised gas/oil pipelines; (b) developing and optimising the data interpretation techniques, particularly 3D spatio-temporal analysis; and (c) integrating syn-collection denoising techniques, such as frequency domain dynamic averaging (FDDA) and/or activation function dynamic averaging (AFDA) methods, to enhance user functionality.

## Figures and Tables

**Table 1:** Operational performance specifications for the Luna ODiSI 6100 dynamic measurement system and pre-calibrated strain sensor.

Luna ODiSI 6100 Measurement System Parameter	Test Configuration	Specification Value	Comment
Strain measurement range	-	±15,000 με	-
Full-range system accuracy	-	±30 με	Instrument and sensor combined
Measurment uncertaincy at zero strain	1.3 mm gauge pitch	±6 με	Instrument and sensor combined
Measurement rate	10 m sensor 1.3 mm gauge pitch	25 Hz	Defined value for dual channel use
HD6S—220C sensor maximum operating temperature	10 m calibrated length	220 °C	-

## Data Availability

The data presented in this study have not been made publicly available due to commercial sensitivity but may be available on request from the corresponding author.
